# Classic Ulcerative Pyoderma Gangrenosum Is a T Cell-Mediated Disease Targeting Follicular Adnexal Structures: A Hypothesis Based on Molecular and Clinicopathologic Studies

**DOI:** 10.3389/fimmu.2017.01980

**Published:** 2018-01-15

**Authors:** Elizabeth A. Wang, Andrea Steel, Guillaume Luxardi, Anupam Mitra, Forum Patel, Michelle Y. Cheng, Reason Wilken, Jason Kao, Kristopher de Ga, Hawa Sultani, Alexander A. Merleev, Alina I. Marusina, Alain Brassard, Maxwell A. Fung, Thomas Konia, Michiko Shimoda, Emanual Maverakis

**Affiliations:** ^1^Department of Dermatology, University of California, Davis, Sacramento, CA, United States; ^2^Department of Pathology, University of California, Davis, Sacramento, CA, United States

**Keywords:** pyoderma gangrenosum, CD4, T cell, autoimmune, cytokine, pilosebaceous unit, IL-36G, pathophysiology

## Abstract

**Background:**

Pyoderma gangrenosum (PG) is a debilitating ulcerative skin disease that is one of the most common associated diseases seen in patients with inflammatory bowel disease and rheumatoid arthritis. Although PG is classified as a neutrophilic dermatosis, its pathophysiology is poorly understood.

**Objective:**

Use data obtained from patient-reported histories, immunohistochemistry, and gene expression analysis to formulate a hypothesis on PG pathophysiology.

**Methods:**

Ten PG patients participated and answered questions about new ulcer formation. Skin biopsies of healed prior ulcers and adjacent normal skin were obtained from four patients for immunohistochemistry. Scars from healthy patients and patients with discoid lupus were used as additional controls. New onset PG papules were analyzed using immunohistochemistry and gene expression analysis *via* quantitative real-time PCR.

**Results:**

All PG patients reported that healed sites of previous ulceration are refractory to re-ulceration. Simultaneous biopsies of healed and uninvolved skin triggered ulceration only in the latter. On immunohistochemistry, healed PG scars showed complete loss of pilosebaceous units, which were present in normal skin, and to a lesser extent in control scars, and discoid scars. Early PG papules showed perivascular and peripilosebaceous T cell infiltrates, rather than neutrophils. These early inflammatory events were dominated by increased gene expression of *CXCL9, CXCL10, CXCL11, IL-8, IL-17*, IFNG, and *IL-36G* and transcription factors consistent with Th1 phenotype.

**Limitations:**

Small sample size was the main limitation.

**Conclusion:**

We put forth the hypothesis that PG is a T cell response resulting in the destruction of pilosebaceous units.

## Nomenclature

PG, pyoderma gangrenosum; H&E, hematoxylin and eosin; HPF, high powered field; qRT-PCR, quantitative real-time PCR; MIQE, Minimum Information for Publication of Quantitative Real-Time PCR Experiments; RIN, RNA integrity number; ANOVA, analysis of variance.

## Introduction

Classic pyoderma gangrenosum (PG) is an ulcerative neutrophilic dermatosis that is the most common skin disease associated with inflammatory bowel disease (IBD) (1–3). It is also one of the more common skin conditions that are frequently comorbid with rheumatoid arthritis (RA), the association of which portends a poor prognosis (4). Diagnosis of PG is extremely challenging and treatment options are limited. Although it is most commonly thought to be a neutrophilic dermatosis, PG pathophysiology is actually poorly understood. The dominant hypothesis is that altered innate immunity leads to systemic autoinflammation (5). This view is supported by the finding that PG and its syndromic form PASH (PG, acne, supparative hidradenitis) are linked to alterations in autoinflammatory genes (6–8). An alternative view is that T cells are involved in PG pathophysiology (9, 10), yet there are no current theories on autoimmune targets.

Despite being difficult to diagnose, PG has distinct features that may provide insight into its pathogenesis. One feature of particular interest is pathergy, the phenomenon of ulcers forming at sites of minor trauma (11). Interestingly, not all sites are susceptible to pathergy. The impetus for the current study came from a PG patient’s observation that PG scars are protected from future ulceration, a finding that is in contrast to ulcers formed from venous stasis or cutaneous vasculitis, in which sites of previous activity are prone to recurrent ulceration.

Herein, we attempt to gain insight into the pathophysiology of PG by characterizing the cellular and molecular events prior to ulcer formation and after ulcer healing. Through our findings, we put forth the hypothesis that PG results from aberrant cytokine expression and autoreactive T cells, possibly directed against pilosebaceous structures. We identify a variety of novel cytokines involved in the process including *IL-8* and *IL-36G*, which can be targets for future drug development for this difficult to treat disease.

## Materials and Methods

### Participants and Design

This study was approved by the UC Davis Institutional Review Board (IRB# 335144). All subjects gave written informed consent in accordance with the Declaration of Helsinki. Ten patients with a history of classic ulcerative PG for their participation were included in this study. Diagnosis of PG was verified by more than one board-certified dermatologist based on clinical history, physical examination findings, and biopsy findings. Specifically, in all cases, the diagnosis of classic ulcerative PG was made and confirmed based on clinical evidence of rapidly progressing painful, necrolytic, and cutaneous ulcer(s) with an undermining violaceous border, as well as exclusion of other causes of cutaneous ulceration (12). In addition, patients had two of the following five characteristics: history of pathergy, clinical evidence of cribriform scarring, systemic diseases associated with PG, compatible biopsy findings, and demonstrated responsiveness to immunosuppression. These criteria are consistent with prior proposed diagnostic criteria (13) as well as the most up-to-date diagnostic criteria (12).

For the patient-reported aspect of this study, all 10 patients were asked about patterns of ulcer formation. Four PG patients with well-controlled disease (i.e., long-term remission on immunosuppressive agents) underwent punch biopsies of PG scars and adjacent normal skin (herein termed “normal skin”) for immunohistochemical analyses. Hypertrophic scars from healthy patients were used as an additional control (herein termed “control scar”). Scars from patients with biopsy-proven discoid lupus were also obtained to additionally control for post-inflammatory changes (herein termed “discoid scar”).

Finally, cytokine/chemokine gene expression analysis was performed on pre-ulcerative PG lesions provided by patient with longstanding history of PG presenting with numerous small papules in an early PG flare.

### Immunohistochemistry

Skin biopsies were paraffin-embedded, and 5 µm sections were stained by Peninsula Histopathology Laboratory Inc. (Campbell, CA, USA). Biopsies were stained with hematoxylin and eosin and Masson’s trichrome. Giemsa stain was used to assess prevalence of mast cells. Immunohistochemical stains were performed with antibodies directed against CD1a (010), CD3, CD4 (4B12), CD8 (C8/144B), CD45RO (UCHL1), CD19 (LE-CD19), CD31 (JC70A), CD34 (QBEND10), CD57 (TB01), CD68 (PG-M1), desmin (D33), smooth muscle actin (1A4), vimentin (V9), factor XIIIa (E980.1), and von Willebrand factor (Peninsula Histopathology Laboratory Inc., Campbell, CA, USA). Slides were assigned histological scores. A blinded dermatopathologist scored each slide [1–10 positive cells/high powered field (HPF) = 1, 11–20 positive cells/HPF = 2, 21–30 positive cells/HPF = 3, and > 30 positive cells/HPF = 4].

### Gene Expression Analysis and Quantitative Real-time PCR (qRT-PCR) Array

Differentially expressed genes of immunologic significance were identified in early pre-ulcerative PG papules vs. normal skin by qRT-PCR array. Tissue samples were stabilized by RNAlater addition (Ambion). Total RNA was extracted using RNeasy plus mini kit (Qiagen), and the quantity and integrity of RNA was determined by fluorometry (Qubit, Thermo Fisher) and 2200 TapeStation, respectively. Total RNA was reverse transcribed to cDNA using iScript (Bio-Rad) and qRT-PCR was performed using customized PrimePCR plates from Bio-Rad with GAPDH, TBP1 and HPRT1 as reference genes following the Minimum Information for Publication of Quantitative Real-Time PCR Experiments (MIQE) Guidelines (14). Samples with RNA integrity number ≥7.5 were used for this study. Using a custom qRT-PCR array with validated primer sets, SsoAdvanced Universal SYBR Green Supermix, and the CFX96 Touch Real-Time PCR Detection System, differential gene expression analysis and corresponding statistical analysis were performed through Bio-Rad CFX Manager software (Bio-Rad Laboratories, Hercules, CA, USA).

### Statistical Analysis

Differences in histological scores between normal skin vs. PG scar vs. control scar vs. discoid scar were assessed with one-way analysis of variance (ANOVA), followed by Bonferroni *post hoc* tests. Bonferroni error correction for multiple comparisons was calculated by dividing an alpha of 0.05 by the number of comparisons (*n* = 6), resulting in an adjustment alpha of 0.0083, which was used as the threshold for statistical significance. SigmaStat 4.0 was used to perform statistical analyses on the histological scores. Bio-Rad CFX Manager was used to perform differential cytokine gene expression analysis and corresponding statistical analysis (Bio-Rad Laboratories, Hercules, CA, USA).

## Results

### Healed PG Scars and Body Areas Devoid of Follicular Adnexal Structures Are Resistant to Development of PG Ulcers

**Figure 1:**
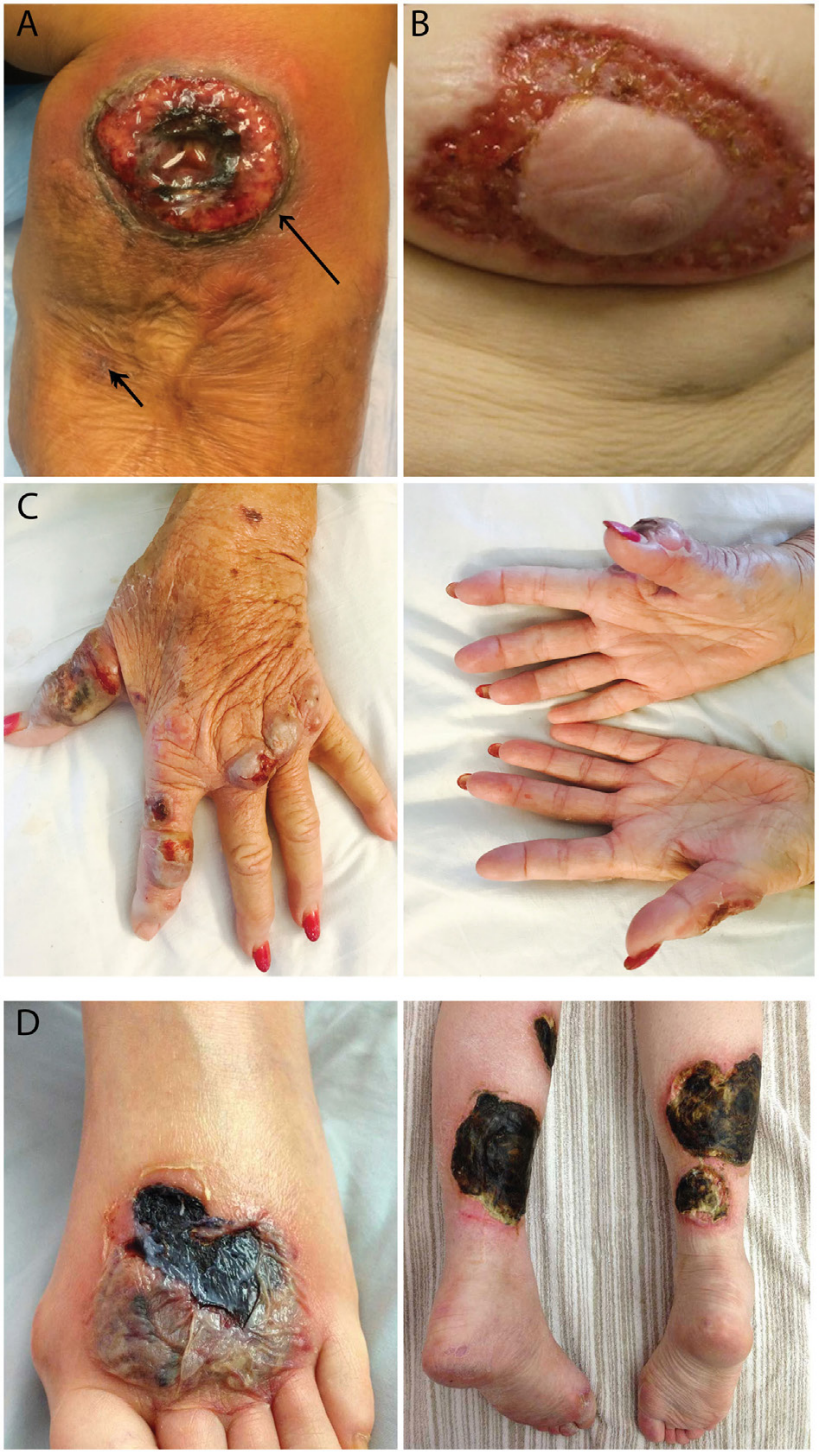
Patterns of ulcer formation in pyoderma gangrenosum (PG). **(A)** PG scars do not re-ulcerate. Two biopsies were obtained simultaneously from a PG patient. Biopsy of a PG scar (short arrow) healed without incident, while biopsy of adjacent normal skin resulted in a new ulcer (long arrow). **(B)** PG ulcers discretely avoid the nipple-areolar complex, an area known to be devoid of pilosebaceous units. **(C)** Hand PG appears to affect only the dorsal surface of the hands. **(D)** A PG lesion affects the dorsal foot and posterior lower extremities, sparing the soles of the feet.

All 10 PG patients reported that new ulcers never occurred at sites of prior ulcerations. In fact, four patients stated that as a PG ulcer expands, it grows to avoid PG scars. This observation was confirmed when a PG patient underwent two biopsies at the same visit (Figure 1A, arrows). The trauma induced from a biopsy of normal skin resulted in a new PG ulcer, but no ulcer formed at the adjacent biopsy site located within a PG scar. This led us to hypothesize that the resistance of PG scars to ulceration may be derived from its lack of an autoantigen target, possibly follicular adnexal structures. In accord with this possibility is the observation that PG ulcers do not appear to develop in body areas lacking follicular adnexal structures and in fact appear to discretely avoid these areas (Figures 1B–D). These protected areas include the nipple-areolar complex (Figure 1B), the palmar hands (Figure 1C), and the soles of the feet (Figure 1D).

### Complete Lack of Pilosebaceous Units and Alterations in Resident Immune Cells in PG

**Figure 2:**
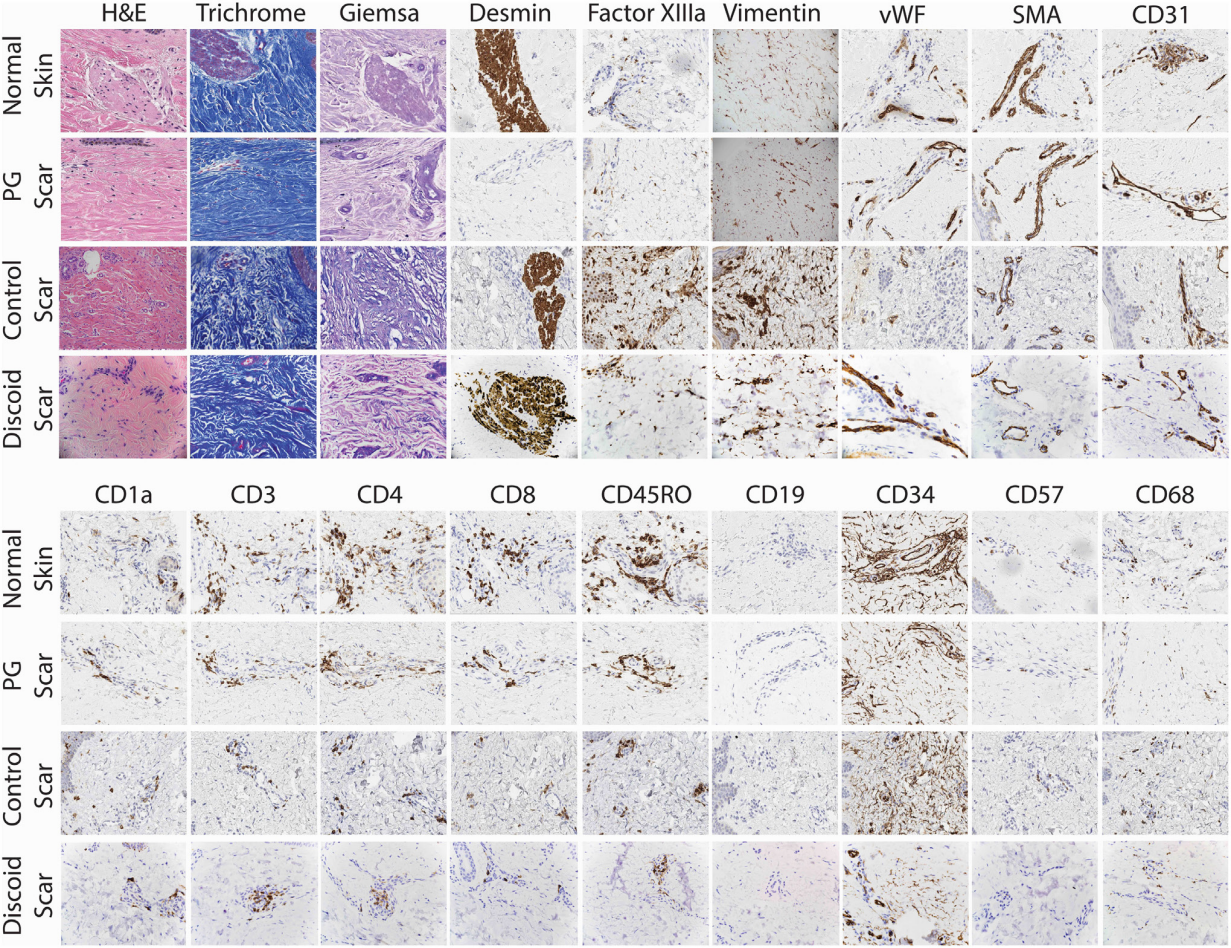
Detailed histological analysis of pyoderma gangrenosum (PG) scars (*n* = 4), adjacent normal skin, control scars, and discoid scars. Under 20× magnification, there was no significant difference between PG scars, normal adjacent skin, and discoid scars in the prevalence of mast cells (Giemsa), fibroblasts (vimentin), myofibroblasts (SMA), endothelial cells (CD31, vWF), macrophages (CD68), T cells (CD3), cytotoxic T cells (CD8), memory T cells (CD45RO), and NK cells (CD57). Adnexal structures are absent in PG scars (desmin) but present in normal adjacent skin, control scars, and discoid scars.

**Figure 3:**
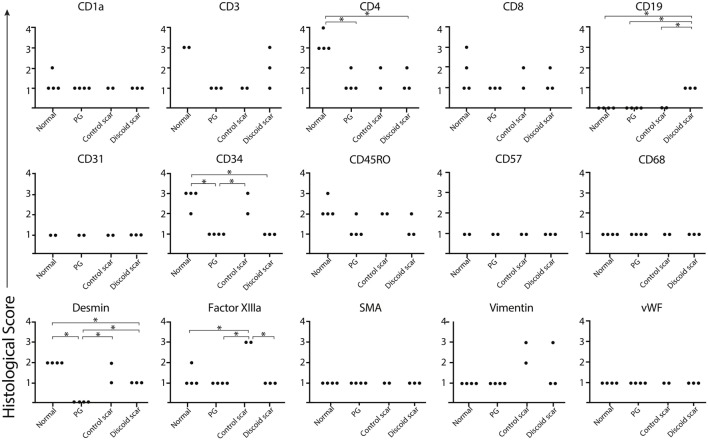
Pyoderma gangrenosum (PG). Scoring diagram. A blinded dermatopathologist scored each slide [1–10 positive cells/high powered field (HPF) = 1, 11–20 positive cells/HPF = 2, 21–30 positive cells/HPF = 3, and >30 positive cells/HPF = 4]. There is significantly less CD4^+^ (*p* = 0.003) and CD34^+^-expressing cells (*p* ≤ 0.001) and undetectable desmin (*p* ≤ 0.001) in a healed PG scar compared to the adjacent normal skin.

To confirm the lack of follicular adnexal structures in PG scars, biopsies of PG scars and adjacent normal skin were obtained for immunohistochemical analysis. This revealed an absence of adnexal structures in PG scars, an observation confirmed by desmin staining, which was undetectable (Figures 2 and 3). Immunohistochemistry revealed that there was a statistically significant difference in histological scores, which were assigned by a blinded dermatopathologist, in normal skin vs. PG scar vs. control scar vs. discoid scar (*p* < 0.001), and Bonferonni *post hoc* tests demonstrated a statistically significant decrease in desmin in PG scars when compared to normal skin (*p* < 0.001), control scars (*p* = 0.001), and discoid scars (*p* = 0.002) (Figure 3). Control and discoid scars predictably had few adnexal structures but were not entirely devoid of them as shown by low but positive residual desmin staining. In contrast to the typically held view that the wrinkled paper appearance of PG scars is a result of skin atrophy, the histologic appearance was not consistent with thinning of the dermis. However, by immunohistochemistry, PG scars did have statistically significantly fewer CD4^+^ (helper) (*p* = 0.003) and CD34^+^ cells (fibroblasts) (*p* < 0.001) (Figure 3). There was also a trend toward differences in CD45RO (memory) T cells by ANOVA (*p* = 0.059) (Figure 3). Both discoid scars and PG scars had significantly decreased CD34^+^ cells (fibroblasts) when compared to normal skin (*p* ≤ 0.001).

### Perivascular and Periadnexal T Cell Infiltration Precede Ulcer Formation

**Figure 4:**
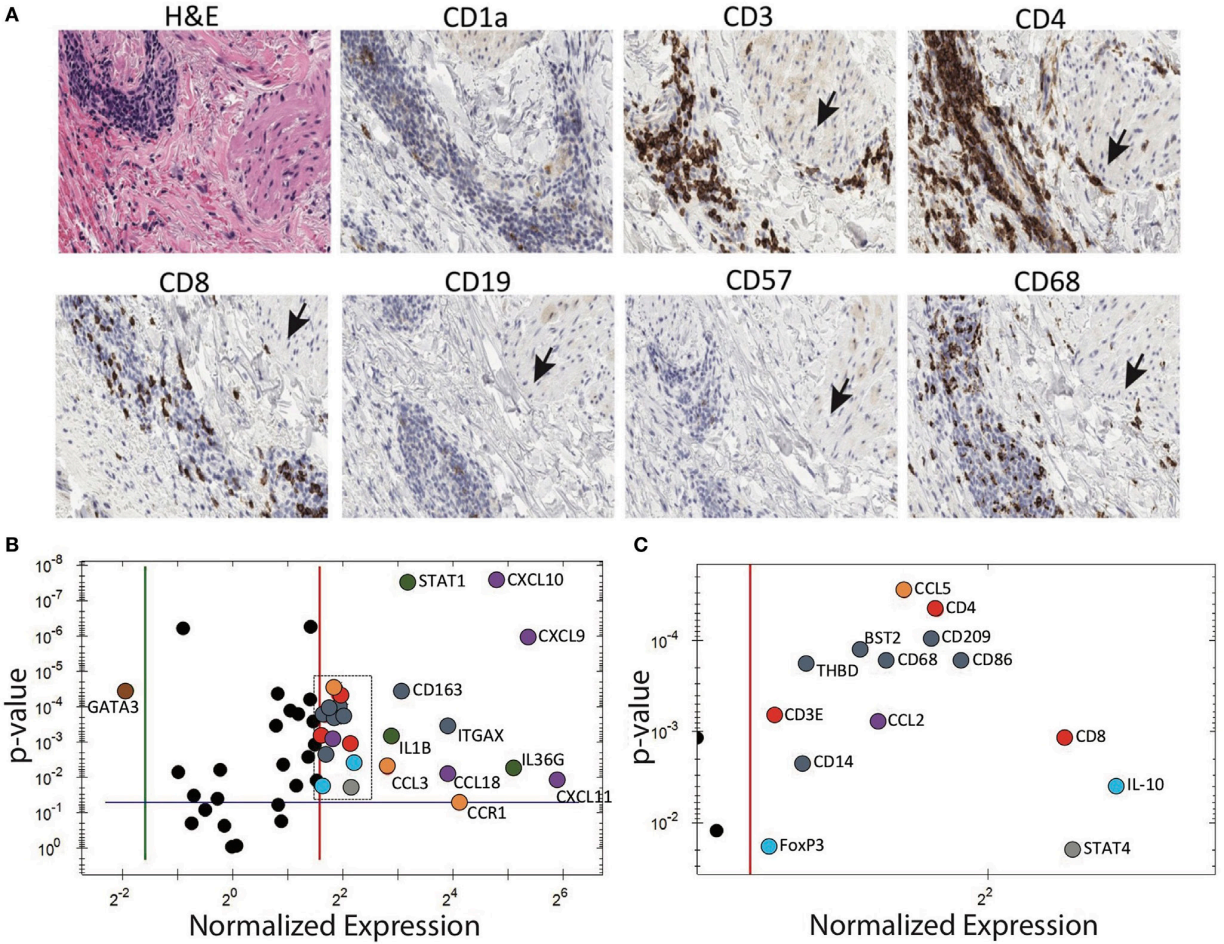
Pyoderma gangrenosum (PG) immunohistochemistry and gene expression analysis. **(A)** Histology and immune markers in early PG papule. There is dense perivascular CD3^+^, CD4^+^, and to a lesser extent CD8^+^ infiltration. The close proximity to a hair follicle is evident by the arrector pili muscle (indicated by arrows). **(B)** Gene expression comparing cytokines in an early PG papule vs. healthy skin. Among the 80 genes studied, the PG papule showed significant (*p* ≤ 0.05) 26 upregulation and 1 downregulation genes compared to healthy skin. In the diagram, genes that are significantly upregulated in PG are subgrouped and differentially colored based on its functions: antigen-presenting cells—dark blue, T cells—red, IL-1 family (including IL-36G)—green, T cell chemoattractants—purple, regulatory T cells—light blue, neutrophil recruitment—orange, and Th1—gray. The top four upregulated gene(s) include CXCL9, CXCL10, CXCL11, and IL36G. IL-8 and IL-17 were also strongly expressed in PG papules, but were undetectable in healthy normal skin, and therefore the ratio cannot be depicted in this diagram. IFNG was also detected in PG papules but absent in normal skin. **(C)** Expanded view of boxed area of panel **(B)**.

A patient with longstanding PG presented to us with numerous small papules. This provided us with the rare opportunity to biopsy very early, pre-ulcerative PG lesions. Subsequently, these papules, both biopsied and unbiopsied papules, rapidly progressed into ulcers consistent with prior episodes of PG. Immunohistochemical examination of the papules revealed a dense perivascular CD3^+^ infiltrate. Deeper sections revealed dense T cell infiltrates centered on/near hair follicles with sparse-to-moderate lymphocytes centered around deeper adnexal structures (Figure 4A).

### Multiple Cytokines Are Highly Expressed in Early PG Lesions

Gene expression analysis was performed to elucidate how T cell-dominant papules evolve into PG ulcers, which on histology are characterized by a neutrophil-dominant or a mixed cellular infiltrate. A qRT-PCR array (80 genes) detected increased expression of cytokines *CXCL9, CXCL10, CXCL11, IL-36G*, and *IL-17A* in early lesional skin compared to healthy normal skin (Figures 4B,C). *IFNG* was also detected in PG lesions but not in adjacent normal skin. In addition, there was overexpression of numerous neutrophil-attracting chemokines, including *IL-8, CCL-3* and *CCL-5* (Figures 4B,C). Importantly, the Th2-promoting transcription factor *GATA3* was strongly downregulated, while the Th1-promoting transcription factors *STAT1* and *STAT4* were highly upregulated (Figures 4B,C; Table 1).

**Table 1:** Differentially expressed genes in early pyoderma gangrenosum (PG) papules with corresponding fold change and *p*-values.

Gene name	Fold change^a^	*p*-Value**
*ITGAX*	15.1	4 × 10^−4^
*CD163*	8.5	3 × 10^−5^
*CD86*	3.9	2 × 10^−4^
*CD209*	3.7	1 × 10^−4^
*CD68*	3.5	2 × 10^−4^
*CD14*	3.2	2 × 10^−3^
*BST2*	3.4	1 × 10^−4^
*THBD*	3.2	2 × 10^−4^
*CD3E*	3.1	7 × 10^−4^
*CD4*	3.8	5 × 10^−5^
*CD8a*	4.4	1 × 10^−3^
*IL-1B*	7.5	7 × 10^−4^
*STAT1*	9.1	3 × 10^−8^
*IL36G*	34.3	6 × 10^−3^
*CXCL10*	27.8	3 × 10^−8^
*CXCL9*	41.6	1 × 10^−6^
*CXCL11*	59.8	1 × 10^−2^
*CCL18*	14.9	8 × 10^−3^
*CCL2*	3.5	8 × 10^−4^
*Foxp3*	3.1	2 × 10^−2^
*IL-10*	4.7	4 × 10^−3^
*CCL3*	7.0	5 × 10^−3^
*CCL5*	3.6	3 × 10^−5^
*CCR1*	17.4	5 × 10^−2^
*STAT4*	4.4	2 × 10^−2^
*GATA3*	−3.8	3 × 10^−5^

*^a^Differential gene expression (PG early papule/non-lesional skin)*.

**Calculated through Bio-Rad CFX Manager software with multiplicity adjusted p-values.

*Of note, genes that are detected in lesional skin but undetectable in normal skin are not listed in this table*.

## Discussion

The main goal of the present study is to shed insight into the immune pathogenesis of PG, a severe ulcerative skin condition that is one of the most common cutaneous manifestations of IBD and RA.

A striking finding evident from our study is that PG scars are refractory to re-ulceration, even in the setting of trauma, as shown in our patients who were biopsied at scar sites without subsequent incident. This is also in contrast to psoriatic lesions and ulcers formed from venous stasis or cutaneous vasculitis where sites of previous activity are prone to recurrent disease (15). PG ulcers also appear to preferentially avoid body areas devoid of follicular adnexal structures, e.g., the nipple-areolar complex and the palms and soles. This is in contrast to Sweet’s syndrome (a neutrophilic disease), scleroderma, discoid lupus, and other autoimmune diseases, which indeed can occur at these sites (16).

Based on our clinical and histologic observations, we put forth the hypothesis that PG may result from an adaptive immune response targeting pilosebaceous units. Our hypothesis would explain why PG scars, by lacking these structures, are resistant to future immunologic insult. The complete absence of pilosebaceous units in PG scars not only appear to confer resistance to future ulceration but also highlight their potential role as an autoantigen target critical to the pathophysiology of PG. The distribution of pustular PG, which is centered on hair follicles, is also evidence to support this hypothesis (17).

Importantly, pilosebaceous units likely serve as autoantigens in other diseases as well. For example, the pilosebaceous unit also appears to be targeted in the setting of discoid lupus, although the cell types and cytokines involved are different. The inflammatory process in discoid lupus can lead to the permanent destruction of pilosebaceous units (18). In severe cases, recurrent disease affects the edges of scarred lesions but not within them (19), which is similar to what we propose to occur in PG.

Adnexal structures are also an important target in the setting of another cutaneous inflammatory disease, lichen planopilaris (LPP), which is characterized by follicular hyperkeratosis, perifollicular erythema, and ultimately destruction and fibrosis of hair follicles. However, even within the scarring patches of LPP, residual small hairy islands can be the focus of active or recurrent disease (20). These examples highlight the importance of adnexal structures as a target in immune-mediated skin diseases and reveal a possible mechanism of disease recurrence and resistance. Interestingly, indicators of increased interferon-mediated signaling (CXCL9, CXCL10, and CXCL11) are also heightened in LPP (21), and scarring skin lesions of discoid lupus are also characterized by high numbers of skin-homing cytotoxic lymphocytes associated with strong expression of Th1 markers (22), although *IL8* and *IL36G* have not been reported to play a role in LPP or discoid lupus. Thus, PG appears to have an overlapping but unique cytokine signature.

An attack against the pilosebaceous units in PG would also explain the characteristic “undermined” border that typifies a PG ulcer. For such a phenomenon to exist, the site of inflammation must reside well below the epidermis. Indeed, when we sampled very early PG papules, immunohistochemical analysis revealed a dense perivascular T cell infiltrate (mainly CD4^+^) that was centered on/near hair follicles with sparse-to-moderate involvement of deeper adnexal structures.

Gene expression analysis complements these findings as our qRT-PCR array of an early PG papules revealed robust expression of T cell attractant chemokines *CXCL9, CXCL10, CXCL11*, and the cytokines *IL8, 17*, and *36G*, the latter of which is known to significantly induce immune cells and keratinocytes to secrete cytokines attracting macrophages, T cells, and neutrophils (23). The initial T cell-dominant infiltrate of PG was mainly associated with Th1 and Th17 cytokines and chemokines, and strong downregulation of the Th2-promoting transcription factor *GATA3*. Together, these findings support T cell-mediated autoimmunity as the cause for PG. This differs from the conventional view that PG is a neutrophil-mediated disease but is consistent with others who have also suggested a role for T cells (9, 24, 25).

These findings further elicit the question: what is the initial immunologic trigger in PG? One hypothesis is that trauma, a known trigger of PG, causes the release of cytokines and danger signals that initiate the aberrant immune response. In fact, trauma-induced release of IL-36 cytokines has been suggested to play an important initiating role in the highly similar Koebner phenomenon of psoriasis (i.e., formation of lesions at sites of injury) (26). Among other pro-inflammatory cytokines, minor trauma to the skin has also been shown to increase IL-8 gene expression. Thus, two of the aberrantly expressed cytokines seen in early PG lesions are known to be associated with trauma. Tissue damage can also cause the release of autoantigens specific to the pilosebaceous unit. This together with the local inflammatory milieu in a genetically susceptible host may be sufficient to induce a T cell dominant infiltrate, resulting in the additional production of pro-inflammatory and neutrophil-recruiting cytokines, ultimately leading to ulcer formation.

The main limitation of our study is the small sample size, mirroring the low incidence of this disease. Also, while PG is one of the most common skin manifestations of IBD and RA, the opportunity that we had to sample pre-ulcerative lesions from a patient who is newly presenting and off immunosuppressive therapy is exceedingly rare. The chance encounter with this patient allowed us to take advantage of a unique window into the earliest pathophysiological events leading to this devastating condition. Since ulcers rapidly form, most lesions are well developed at the time of presentation.

Although further studies will be required to validate our findings, historically, the role of follicular structures in PG was noted by the late dermatopathlogist Dr. Ackerman (27) and is strongly supported by the clinical distribution of PG in which the nipple-areolar complex, palms, and soles are spared. These sites are absent in follicular adnexal structures and are in fact not spared in other autoimmune diseases such as scleroderma and discoid lupus. They are also not spared in other neutrophilic diseases such as Sweet’s syndrome.

While the role of acquired immunity in the pathogenesis of PG remains poorly investigated, our findings demonstrate that the initial inflammatory events of PG are dominated by aberrant cytokine and chemokine expression and by perivascular and perifollicular T cells. These findings add to the existing literature suggesting that T cells play a contributing role in the pathogenesis of the disease. We thus put forth the hypothesis that PG results from aberrant cytokine expression and autoreactive T cells directed against follicular adnexal structures.

## Ethics Statement

This study was approved by the UC Davis Institutional Review Board (IRB# 335144). All subjects gave written informed consent in accordance with the Declaration of Helsinki.

## Author Contributions

EM had full access to all of the data in the study and takes responsibility for the integrity of the data and the accuracy of the data analysis. Study concept and design: EM, EW, AS, AM, FP, MC, and RW. Acquisition, analysis, and interpretation of data: EM, EW, AS, AM, FP, KG, JK, RW, MS, AB, GL, TK, MF, HS, and MC. Drafting of the manuscript: EM, EW, AS, AM, FP, and MC. Critical revision of the manuscript for important intellectual content: EM, EW, AS, MC, MF, TK, HS, AB, MS, GL, AAM, and AIM. Statistical analysis: EM, EW, JK, AM, and TK. Obtained funding: EM. Administrative, technical, or material support: EM, EW, JK, KG, HS, and MY. Study supervision: EM. Final approval of the version to be published: EM, EW, AS, AM, FP, MC, KG, JK, RW, MS, AB, GL, TK, MF, and HS.

## Conflict of Interest Statement

The authors declare that the research was conducted in the absence of any commercial or financial relationships that could be construed as a potential conflict of interest.
